# Neural Structures within Human Meniscofemoral Ligaments: A Cadaveric Study

**DOI:** 10.1155/2014/719851

**Published:** 2014-03-10

**Authors:** Chinmay M. Gupte, Daniel A. Shaerf, Ann Sandison, Anthony M. J. Bull, Andrew A. Amis

**Affiliations:** ^1^Department of Mechanical Engineering, Imperial College London, London SW7 2AZ, UK; ^2^Musculoskeletal Surgery Group, Department of Surgery and Cancer, Imperial College London, Charing Cross Hospital, London W6 8RF, UK; ^3^Imperial College NHS Trust, London W2 1NY, UK; ^4^Pathology Department, Charing Cross Hospital, London W6 8RF, UK; ^5^Department of Bioengineering, Imperial College London, London SW7 2AZ, UK

## Abstract

*Aim.* To investigate the existence of neural structures within the meniscofemoral ligaments (MFLs) of the human knee. *Methods.* The MFLs from 8 human cadaveric knees were harvested. 5 *μ*m sections were H&E-stained and examined under light microscopy. The harvested ligaments were then stained using an S100 monoclonal antibody utilising the ABC technique to detect neural components. Further examination was performed on 60–80 nm sections under electron microscopy. *Results.* Of the 8 knees, 6 were suitable for examination. From these both MFLs existed in 3, only anterior MFLs were present in 2, and an isolated posterior MFL existed in 1. Out of the 9 MFLs, 4 demonstrated neural structures on light and electron microscopy and this was confirmed with S100 staining. The ultrastructure of these neural components was morphologically similar to mechanoreceptors. *Conclusion.* Neural structures are present in MFLs near to their meniscal attachments. It is likely that the meniscofemoral ligaments contribute not only as passive secondary restraints to posterior draw but more importantly to proprioception and may therefore play an active role in providing a neurosensory feedback loop. This may be particularly important when the primary restraint has reduced function as in the posterior cruciate ligament—deficient human knee.

## 1. Introduction

The knee joint is stabilised by passive restraints, such as the capsule and ligaments, as well as active restraints. It has been suggested that the ligaments of the knee may contribute to active stability by providing proprioceptive input to the nervous system, which in turn would adjust muscle contraction accordingly [1]. The anatomy of the meniscofemoral ligament (MFL) has previously been described [2]; the femoral origins of the anterior MFL are distal to the PCL, close to the articular cartilage, whereas the posterior MFL arises proximal to the PCL. They are both inserted distally to the posterior horn of the lateral meniscus. It has been shown that the mechanical role of the meniscofemoral ligament (MFL) is to resist anteroposterior and rotatory laxity in the knee. This is the most important when the primary stabiliser of posterior laxity, the posterior cruciate ligament, is deficient [3].

Proprioceptive nerve endings were initially thought to be located in muscles (as muscle spindles) [4]. More recently, mechanoreceptors have been found in the cruciate ligaments of both animals and humans [1, 5–7]. Kennedy et al. found mechanoreceptors within multiple clefts at the tibial attachment of the anterior cruciate ligament (ACL), within the vascular synovial covering [8]. Schultz et al. reported the presence of mechanoreceptors at the surface of human cruciate ligaments, just beneath the synovial covering [1]. The mechanoreceptors were 200 um long and 75 um wide and resembled Golgi tendon organs. However, there were no receptors in the joint capsule or the two menisci. Schutte et al. also demonstrated mechanoreceptors that morphologically resembled Ruffini endings, Golgi tendon organs, and Pacinian corpuscles, which were predominantly present near the tibial attachment [5]. More recently Pacinian corpuscles and free nerve ending type mechanoreceptors have been found in the posterior septum of the knee [9].

Despite studies demonstrating mechanoreceptors in the posterior cruciate ligament (PCL) of the human knee [1, 7, 10, 11], there are no accounts investigating the presence of mechanoreceptors in human MFLs. O'Connor demonstrated the presence of mechanoreceptors in the meniscofemoral and meniscotibial portions of the canine lateral meniscus [6]. He described type II and type III endings, according to the Freeman and Wyke classification (1967), which were present mainly in the meniscal (distal) portion of the MFL. The presence of mechanoreceptors in the MFL lends weight to the hypothesis that these structures may provide sensory information from the knee which may form part of a protective reflex loop [12].

Noting the presence of mechanoreceptors in the cruciate ligaments and that the MFLs act in synergy with the PCL to stabilise the knee [3], it was hypothesised that the MFLs would also contain mechanoreceptors.

## 2. Aim

The purpose of this study was to ascertain whether there are any neural structures in the MFL of the adult human knee.

## 3. Materials and Methods

Eight cadaveric knees were harvested from four donors after informed consent and ratification from the local hospital ethics board. The mean age of these donors was 76 years. Two of these specimens exhibited gross arthritis and were excluded from the study. The specimens were fresh frozen immediately after harvest. Examination took place 3–9 months after freezing.

After removal of the patellar tendon, ACL, collateral ligaments, and posterior capsule, the anterior and posterior surfaces of the PCL were inspected for the presence of the MFLs. The distal attachment of the MFL was removed by detaching the lateral meniscus from the tibia. The distal attachment of the PCL was removed from each specimen by detaching the PCL close to the bone. As both structures attach proximally into the lateral aspect of the medial femoral condyle, they were detached from this area as close to bone as possible. The specimens were debrided of all extraneous material, such as fat, but no attempt was made to remove the adherent synovial tissue. This ensured that the PCL and MFLs were preserved without damage to their substance.

The specimens were stored in 10% buffered formalin until being transferred to the laboratory. Longitudinal sections were obtained from the tibial and femoral ends of the PCL and the meniscal attachment and femoral end of the MFLs. These specimens were marked with India ink and labelled so that it was possible to identify approximately the location of any neural structures present.

The presence of calcium was detected using Faxitron X-ray. If necessary, the samples were decalcified in 10% formic acid. All samples were dehydrated in a series of ethanols and were then processed to paraffin wax. Longitudinal serial 5 *μ*m sections were prepared using a standard AO rotating microtome. Routine staining was performed using haematoxylin and eosin. The prepared slides were studied under the light microscope.

Antibody staining was performed on paraffin sections using the standard avidin-biotin complex (ABC) method. Before the ABC method could be applied, all slides required “blocking” to prevent background staining. In this process, a solution of hydrogen peroxide was used to remove any endogenous peroxidase enzyme present in the sample, which can be associated with erythrocytes and leucocytes. In the present study the primary antibody was rabbit anti-human polyclonal antibody (DAKO AS, Denmark) and the secondary antibody was swine anti-rabbit antibody.

Each slide was exposed to the primary antibody for 60 minutes. The sections were then washed in Tris buffered saline (TBS) and the biotinylated secondary antibody was applied for 35 minutes. These slides were rinsed in TBS and exposed to the ABC complex for 5 minutes. The use of swine antibody on human tissue can result in immune cross-reactivity of the secondary antibody with endogenous immunoglobulins; a 10% concentration of nonimmune swine serum was applied in order to minimise cross-reactivity.

Standard positive and negative controls were used in the study. The positive control consisted of standard schwannoma neural tissue. In the negative controls the tissue was processed without exposure to the primary antibody.

## 4. Transmission Electron Microscopy

Tissue from an area adjacent to that used for light microscopy was processed from paraffin wax for transmission electron microscopy (TEM). Semithin sections (approximately 1 *μ*m thick) were stained with toluidine blue for light microscopic examination to determine the area of the sample under examination. Ultrathin sections (60–80 nm) were cut and stained with uranyl acetate and Reynold's lead citrate for examination by TEM. TEM was used to identify the presence of neural structures in the MFLs. Quantification of the different types of end-organs found in the specimens was beyond the scope of this study. Tissue from live human sural nerve was used as a standard control for the TEM study. This was analysed in a similar manner to that from the cadaveric MFL.

## 5. Results

### 5.1. Gross Anatomy

Of the 6 specimens suitable for the study, both MFLs coexisted in 3. In 2 specimens, only the anterior meniscofemoral ligament (aMFL) was present and in 1 specimen only the posterior meniscofemoral ligament (pMFL) was present.

### 5.2. Haematoxylin and Eosin Staining

**Figure 1:**
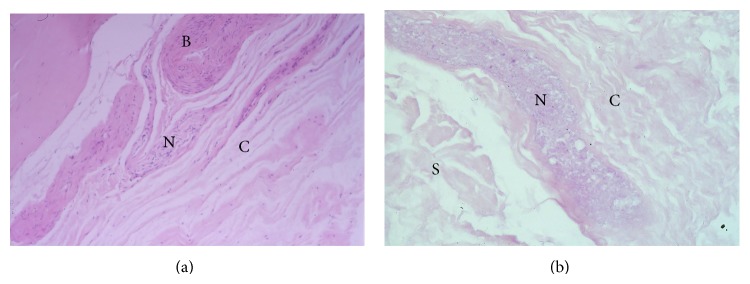
(a) Haematoxylin and eosin staining (×810) of the tibial end of a PCL, showing a structure morphologically consistent with neural tissue (N) adjacent to collagen fibres (C) and blood vessels (B). (b) Haematoxylin and eosin stain (×370) of the meniscal end of an aMFL, showing a structure morphologically consistent with a nerve (N) adjacent to collagen fibres (C) and synovium (S). In this specimen the nerve was in the substance of the ligament close to the synovial surface.

Four of 6 PCL specimens demonstrated structures that were morphologically consistent with nerve fibres (Figure 1(a)), whilst 4 of the 9 MFL specimens—2 of the aMFLs and 2 of the pMFLs—demonstrated such structures (Figure 1(b)). These structures showed degenerative changes consistent with postmortem autolysis, that is, indistinct nuclei and blurring of the tissues. Perineurium was identified, together with the “wavy” (serpiginous) morphology associated with neural structures.

### 5.3. S100 Staining

**Figure 2:**
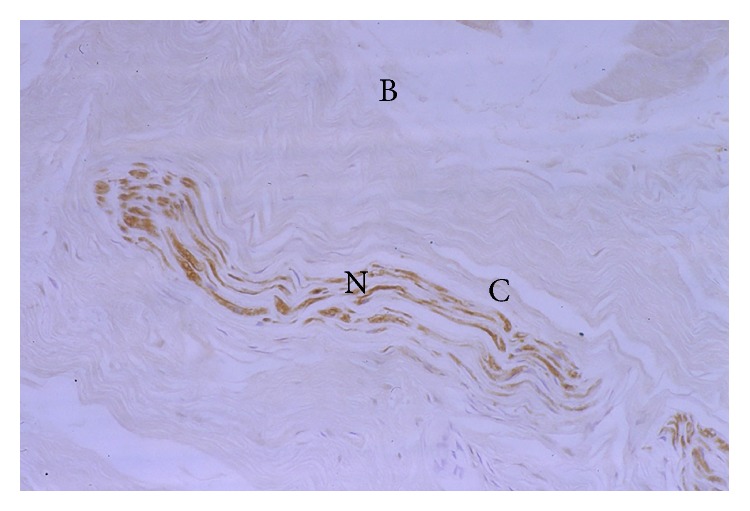
S100 positive immunostaining (brown) in the substance of the tibial end of the PCL; ×700.

**Figure 3:**
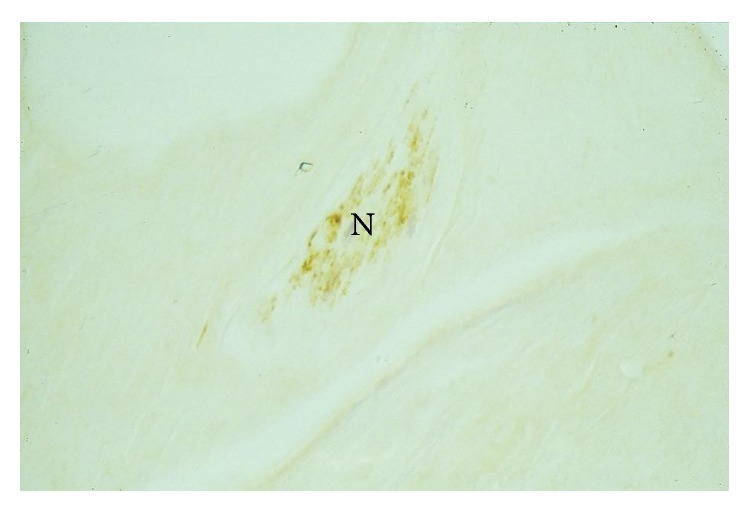
S100 positive immunostaining in the substance of the meniscal end of a pMFL. This structure was morphologically consistent with a nerve (N) ×450.

**Figure 4:**
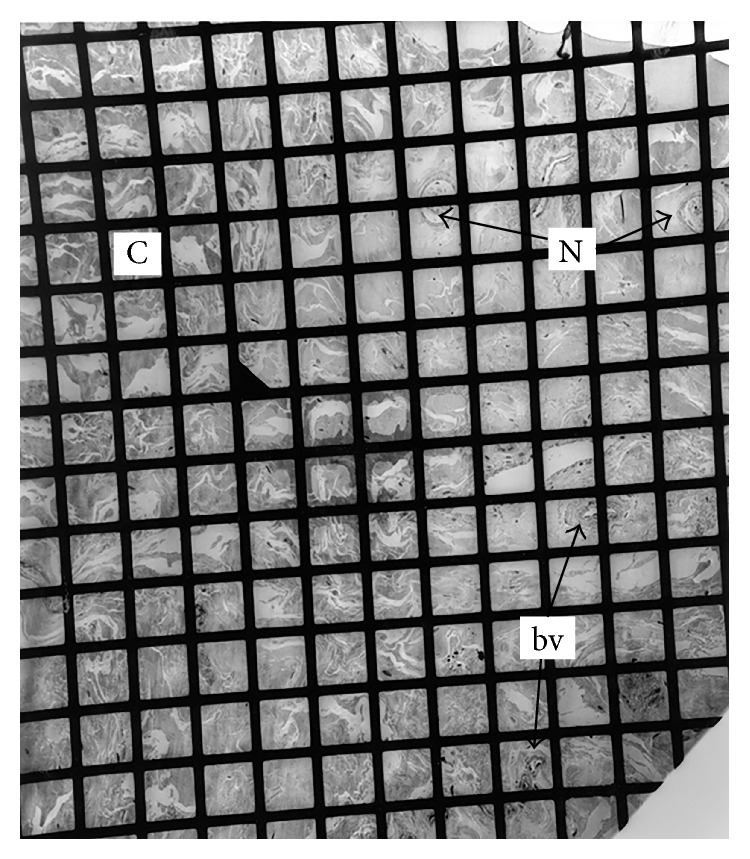
Low magnification transmission electron micrograph of the meniscal end of an aMFL. The sample consisted of bundles of banded collagen fibrils (C) interspersed between blood vessels (bv). Two structures (N) were present which were composed of fibrous sheaths surrounding small cytoplasmic processes interspersed between banded collagen. These structures had the general morphological appearance of nerves. The space between grid lines represents 52.5 um.

The standard negative controls showed no staining with the S100 stain, whilst the standard positive controls stained strongly positively with S100 stain. The S100 stain was positive (as evidenced by brown staining) in 4 of the 6 PCL specimens (Figure 2) and 4 of the 9 MFL specimens (Figure 3). In general, those neural structures within the MFLs were identified at the meniscal (distal) end of these ligaments, whilst those in the PCL were identified near its tibial attachment. Most receptors were present in the synovium surrounding the ligaments. In two specimens, mechanoreceptors were found in the substance of the MFL close to the synovial surface.

### 5.4. Electron Microscopy

TEM of the control neural tissue confirmed the presence of structures morphologically consistent with encapsulated nerve fibres on low magnification. Electron microscopy confirmed the presence of structures morphologically consistent with nerves in the PCL and both MFLs of those ligaments that stained positively with the S100 stain (Figure 4). These structures were close to blood vessels. Although there were structures morphologically similar to nerves, features such as Schwann cells and external laminae were not seen (see Discussion).

**Figure 5:**
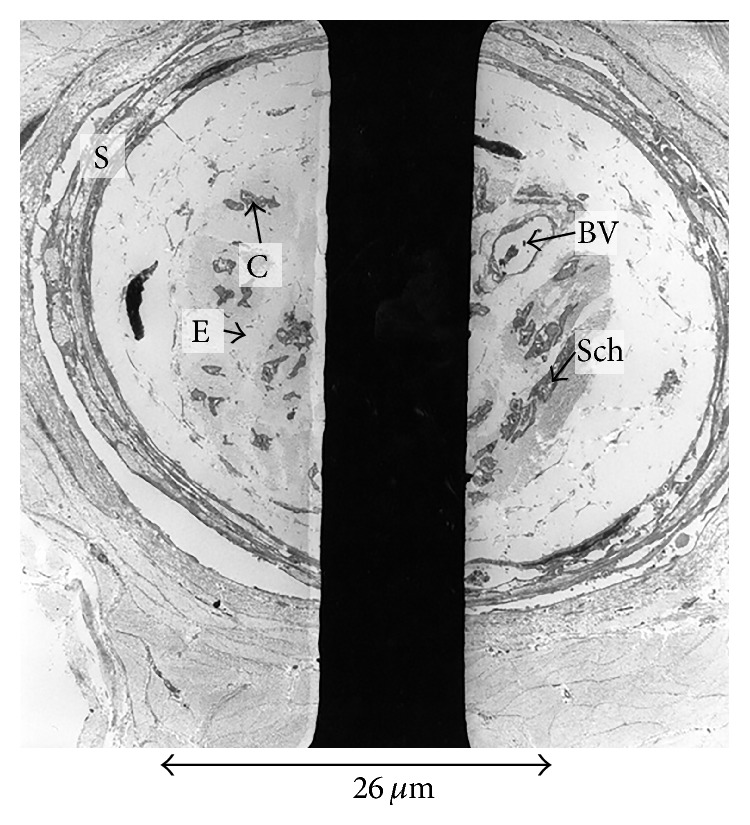
Electron micrograph of the meniscal end of an aMFL, showing a structure morphologically consistent with neural tissue, as evidenced by a perineural sheath (S), surrounding endoneurium (E) and collagen (C). There is also a capillary (BV), together with Schwann cell fragments (Sch).

**Figure 6:**
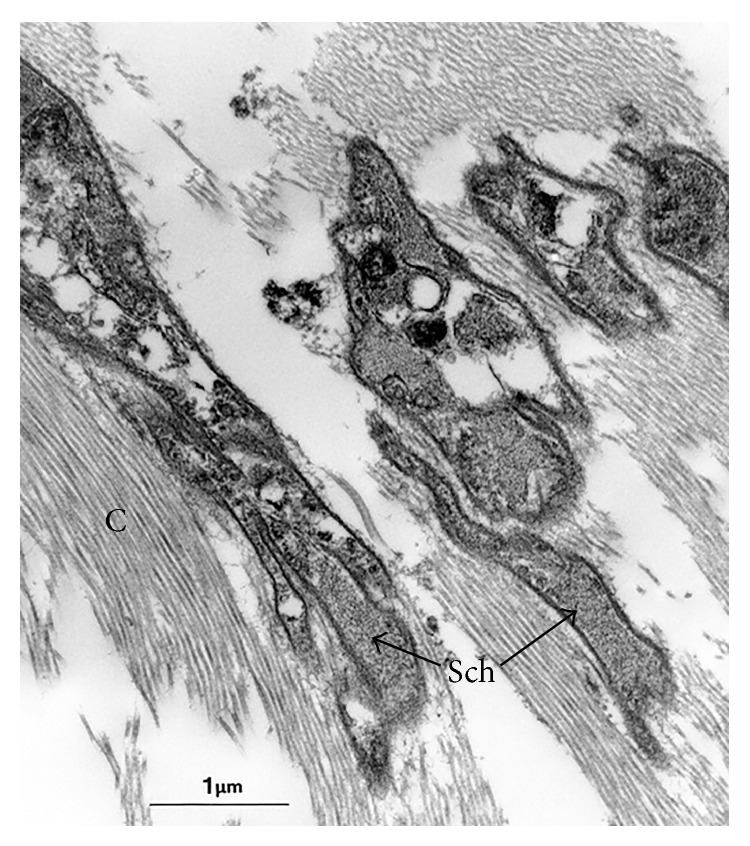
Electron micrograph of meniscal end of a pMFL, showing structures morphologically consistent with Schwann cell cytoplasm (Sch) interspersed with collagen fibrils (C). However, no external lamina was seen, probably due to the processing and degenerate nature of the cadaveric tissue.

Ultrastructurally, encapsulated nerve endings were found in both the anterior and posterior MFLs (aMFL and pMFL) (Figures 5 and 6), as well as in the PCL. It was not possible to characterise the type of nerve ending present from the sections available. In those PCL and MFL specimens that were positive for neural structures it was qualitatively noted that these structures existed in close proximity to blood vessels, possibly in keeping with neurovascular bundles on the macroscopic scale.

## 6. Discussion

This study confirmed the presence of neural structures suggestive of mechanoreceptors in the PCL, aMFL, and pMFL of cadaveric knees. Of the 6 PCLs examined, 4 were positive for S100 staining, and 4 of the 9 MFLs were positive. The TEM studies further confirmed the presence of structures morphologically consistent with nerves.

The findings of this study were limited because the quality of the samples had been affected. The cadaveric, frozen nature of the specimens is likely to have resulted in degeneration and neurolysis, so affecting the histological analysis and hampering detection and accurate characterisation of the nerve endings. Other histological methods have been used to examine mechanoreceptors, including gold chloride staining [5, 11] and neurofilament protein antibody [10], and could be considered in future studies. The TEM specimens were processed directly from paraffin wax, which would have resulted in poor ultrastructural preservation of the neural structures present. This was demonstrated by the absence of characteristic ultrastructural features, such as intact Schwann cells with external laminae or myelin.

Thus it was not possible to characterise the neural structures as specific types of mechanoreceptors. However, the study suggests that such structures exist in the MFLs. In their immunohistochemical study of the PCL, del Valle et al. found types I and III endings according to the Freeman and Wyke classification [10, 12]. Further studies are required on* ex vivo* MFLs (obtained from procedures such as knee arthroplasty) that are processed immediately after being harvested with glutaraldehyde fixation, postfixed with osmium tetroxide and araldite embedding for ultrathin sections, to assess whether similar structures are present.

The findings of this study are in line with previous work. Schultz et al. reported that most of the mechanoreceptors were present near the surfaces of the cruciate ligaments. They suggested that such a location allows greater sensitivity of the receptors to ligament deformation [1]. The concentration of the neural structures near the meniscal end of the MFLs is consistent with the canine study of O'Connor [6].

### 6.1. Neural Elements in the MFLs

Previous studies have suggested that such receptors are present in the meniscofemoral part of the canine lateral meniscus [6] and that they are situated to respond to extension of the knee in this species. The current study confirmed the presence of structures morphologically consistent with neural tissue, which stained positively to S100 monoclonal antibody. These are likely to be nerve fibres or nerve endings. Further studies could characterise the type of mechanoreceptor or neural tissue present, their relative density, and their functional significance.

There are several hypotheses as to the significance of mechanoreceptors in knee ligaments. Palmer demonstrated the reflex contraction of the medial hamstrings and vastus medialis in response to stimulation of the deep capsular portion of the medial collateral ligament [13]. The demonstration of mechanoreceptors, together with the significance of proprioception during rehabilitation, has renewed interest in the neurological function of knee ligaments. Several animal studies have shown that mechanoreceptors in joints initiate a reflex contraction of muscle that is thought to be protective to the knee [6].

### 6.2. Hypotheses on the Sensory Role of the MFLs

The presence of neural structures suggestive of mechanoreceptors in the human MFLs demonstrated in this study may have a bearing on the prognosis and management of PCL injuries. Clancy et al. showed that patients with PCL rupture have a reduced posterior drawer if their MFLs had remained intact [14]. Ahn et al. also noted the significance of preserving the PCL remnant and MFLs during PCL reconstruction [15]. This may be due to a role for the MFLs as passive secondary restraints to posterior drawer [3] but may also be due to an active role through a neurosensory feedback loop.

**Figure 7:**
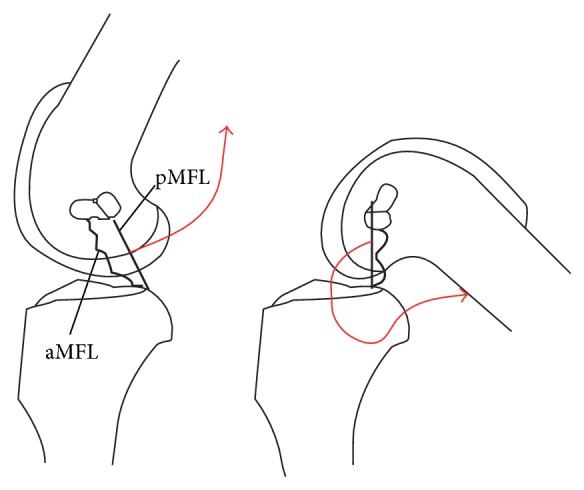
Schematic representation of the reciprocal tension/proprioception hypothesis. Knee extension causes tension in the pMFL (a), whilst the aMFL is taut in flexion (b). If the tightening causes increased afferent activity from the mechanoreceptors in the MFLs (red arrows), it would provide a proprioceptive mechanism.

O'Connor suggested that, in the canine stifle, which has a posterior meniscofemoral attachment, it is taut in extension and such a position would trigger the MFL mechanoreceptor to fire, thus performing a proprioceptive function [6]. In the human, Amis et al. noted that the aMFL is tense in flexion, whilst the pMFL is tense in extension (Figure 7), and so they might have a proprioceptive role in the human knee [2]. It may be that a tight pMFL causes the meniscus to be pulled up towards the femur in hyperextension and therefore be more liable to injury in sudden flexion with internal rotation. This would lead to the posterior horn of the meniscus being ‘squeezed' and prone to tearing. This may form part of a sensorimotor feedback loop leading to external rotation of the tibia.

In the human, Adachi et al. have shown that the number of mechanoreceptors present in ACL remnants of patients undergoing ACL reconstruction correlated well with their preoperative joint position sense [16]. The authors suggested that it might be of value to retain the ACL remnant during reconstruction in order to optimise postoperative proprioception and thereby aid rehabilitation. Other authors have recommended the retention of the PCL during knee arthroplasty, as the proprioceptive function provided by the mechanoreceptors in the PCL may be of value during rehabilitation [10]. The MFLs may provide an additional proprioceptive role.

Several authors have proposed that the isolated rupture of the PCL can be managed nonoperatively with rehabilitation without the need for surgical reconstruction [17–19]. The proprioceptive feedback provided by the intact MFLs may contribute to the success of such management. At present, the MFLs are sacrificed during surgical reconstruction of the PCL when the femoral tunnel is drilled for graft insertion. If the MFLs do provide a proprioceptive role in rehabilitation, it may be of value to develop techniques of preserving the MFLs during PCL reconstruction.

Previous authors have described the proximity of blood vessels to the mechanoreceptors [11]. The present study demonstrated a similar relationship in the MFLs. This may form the basis of neural control of vasomotor responses in this region.

## 7. Conclusion

This is the first study to demonstrate the presence of structures suggestive of neural tissue in the human MFLs. These neural structures were present predominantly in the meniscal (distal) attachments of the MFLs. Thus, it is possible that the MFLs have a proprioceptive function.
